# Data of ecoenzyme activities in throughfall and rainfall samples taken at five subtropical forests in southern China

**DOI:** 10.1016/j.dib.2019.103906

**Published:** 2019-08-29

**Authors:** Taiki Mori, Senhao Wang, Wei Zhang, Jiangming Mo

**Affiliations:** a Key Laboratory of Vegetation Restoration and Management of Degraded Ecosystems, South China Botanical Garden and Guangdong Provincial Key Laboratory of Applied Botany, Chinese Academy of Sciences, Guangzhou, 510650, China; b Department of Forest Site Environment, Forestry and Forest Products Research Institute, Matsunosato 1, Tsukuba, Ibaraki, 305-8687, Japan; c University of Chinese Academy of Sciences, Beijing, 100049, China

## Abstract

The data presented in this article are referred to the research article “A potential source of soil ecoenzymes: From the phylllosphere to soil via throughfall” (Mori et al., 2019). The data included the activities of β-1,4-glucosidase (BG, EC 3.2.1.21), β-d-cellobiosidase (CBH, EC 3.2.1.91), β-1,4-N-acetyl-glucosaminidase (NAG, EC 3.2.1.52), leucine amino peptidase (LAP, EC 3.4.11.1), polyphenol oxidase (PPO, EC 1.10.3.2), and phosphomonoesterase (PME, EC 3.1.3.2). The informatin of study sites and sampling method are shown in Fig. 1 and 2.

**Table:** Specifications Table

Subject area	*Ecology*
More specific subject area	*Ecosystem Ecology*
Type of data	*Table and csv file*
How data was acquired	Fluorescence enzyme assay and spectrophotometric assay.
Data format	*Raw, analyzed*
Experimental factors	*Throughfall and rainfall samples were taken in five types of subtropical forests located in southern China. The samples were taken after rainfall events as soon as possible.*
Experimental features	*A portion of the collected samples was incubated with artificial substrates. After the incubation, we determined the changes in the substrates, which represent the enzyme activity of the collected samples.*
Data source location	*The studied area is situated within Dinghushan Biosphere Reserve (DHS) (112°10′E, 23°10′N), and Heshan National Field Research Station (HS) (112°50′E, 22°34′N) in Guangdong province, China.*
Data accessibility	*Data are available in this article.*
Related research article	*Taiki Mori, Senhao Wang, Wei Zhang, and Jiangming Mo 2019. A potential source of soil ecoenzymes: From the phyllosphere to soil via throughfall. Applied Soil Ecology.*https://doi.org/10.1016/j.apsoil.2019.02.004[1].

**Table:** 

**Value of the data** • This data, for the first time, provide information of ecoenzymes transferred from forest canopy into soils via throughfall. • This data will be available for future works comparing the amount of ecoenzymes in throughfall among different types of forests in different climate zone. • The present data can be reused in future works looking for the contribution of ecoenzyme in throughfall to soil enzyme activity.

## 1 Data

The six types of ecoenzymes, i.e., β-1,4-glucosidase (BG, EC 3.2.1.21), β-d-cellobiosidase (CBH, EC 3.2.1.91), β-1,4-N-acetyl-glucosaminidase (NAG, EC 3.2.1.52), leucine amino peptidase (LAP, EC 3.4.11.1), polyphenol oxidase (PPO, EC 1.10.3.2), and phosphomonoesterase (PME, EC 3.1.3.2) are shown in Table 1. Supplementary csv file provides the raw data of the enzyme activities. The activities of BG, CBH, NAG, LAP, and PME were measured by fluorescence enzyme assays. The activity of PPO was determined by spectrophotometric assay.

**Table 1:** The enzyme activity of throghfall and rainfall in five subtropical forests in southern China.

TF/RF	site	Forest type	BG (μmol MUB h^−1^ L^−1^)		CBH (μmol MUB h^−1^ L^−1^)		LAP (μmol MUC h^−1^ L^−1^)		NAG (μmol MUB h^−1^ L^−1^)		PME(μmol MUB h^−1^ L^−1^)		PPO (μmol DOPA h^−1^ L^−1^)	
			Average	SE	Average	SE	Average	SE	Average	SE	Average	SE	Average	SE
TF	HS	AA	2.316	0.385	0.228	0.078	0.040	0.030	3.058	1.103	9.382	2.056	0.168	0.046
TF	HS	EU	5.809	0.901	0.751	0.209	0.051	0.035	3.534	0.510	10.873	2.773	0.251	0.037
TF	DHS	MF	0.573	0.225	0.039	0.018	0.047	0.013	0.275	0.133	0.437	0.181	0.042	0.024
TF	DHS	BF	0.268	0.096	0.015	0.010	0.043	0.012	0.089	0.050	0.353	0.243	0.009	0.006
TF	DHS	PM	0.342	0.069	0.003	0.003	0.056	0.012	0.074	0.035	0.067	0.044	0.021	0.006
RF	DHS		0.051	0.051	0.033	0.033	0.000	0.000	0.092	0.066	0.067	0.034	0.003	0.003
RF	HS		0.000	0.000	0.000	0.000	0.003	0.003	0.000	0.000	0.000	0.000	0.040	0.006

BF, primary monsoon evergreen broadleaf forest. MF secondary mixed pine/broadleaf forest. PM, planted *Pinus massoniana* forest. AA, planted *Acacia auriculiformis* forest. EU planted *Eucalyptus urophylla* forest. DHS, Dinghushan. HS, Heshan. TF, throughfall. RA, rainfall. BG, β-1,4-glucosidase. CBH, β-d-cellobiosidase. NAG, β-1,4-N-acetyl-glucosaminidase. LAP, leucine amino peptidase. PME, phosphomonoesterase. PPO, polyphenol oxidase.

## 2 Experimental design, materials, and methods

### 2.1 Study site

**Fig. 1:**
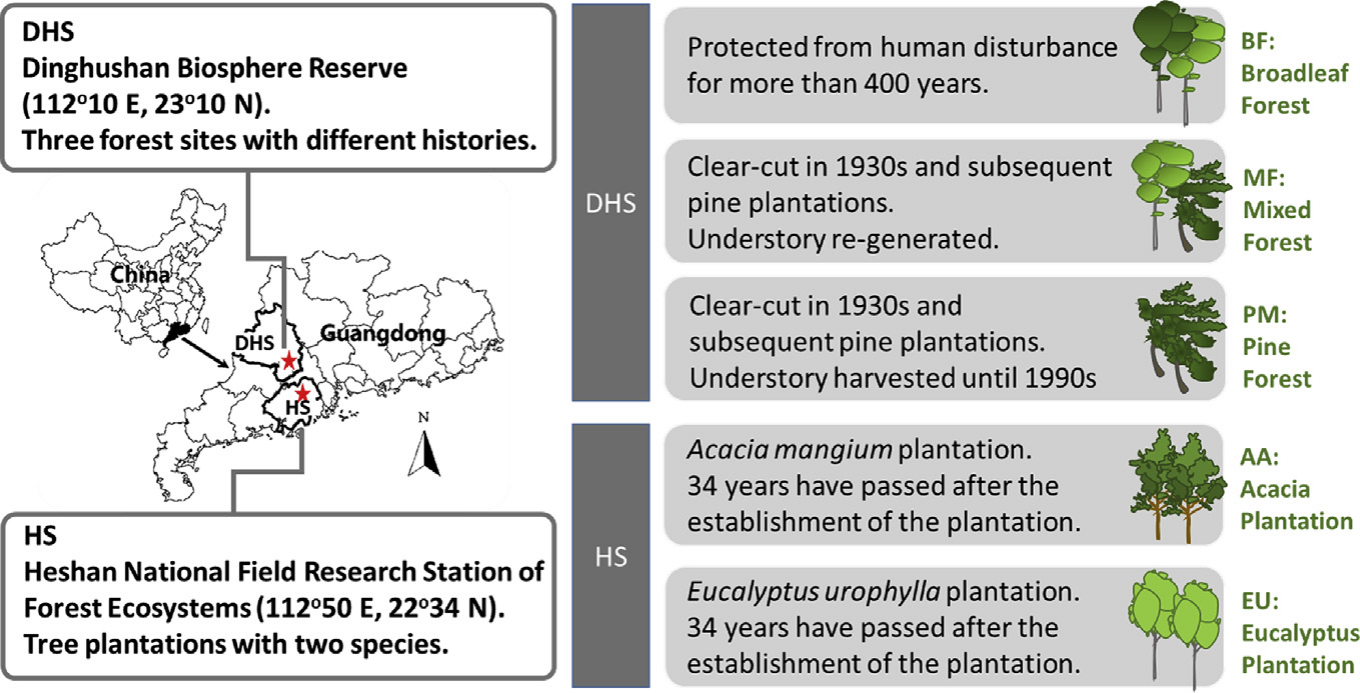
Five subtropical forests located in Guangdong province, China. BF, primary monsoon evergreen broadleaf forest. MF secondary mixed pine/broadleaf forest. PM, planted *Pinus massoniana* forest. AA, planted *Acacia auriculiformis* forest. EU planted *Eucalyptus urophylla* forest. DHS, Dinghushan. HS, Heshan.

This study was conducted in five subtropical forests in two research stations, i.e., Dinghushan Biosphere Reserve (DHS) (112°10′E, 23°10′N) [2] and Heshan National Field Research Station (HS) (112°50′E, 22°34′N) (Fig. 1) [3]. Three types of forests, i.e., a primary monsoon evergreen broadleaf forest (BF), a secondary mixed pine/broadleaf forest (MF), a planted *Pinus massoniana* forest (PM) are located in DHS. According to earlier studies, the BF has been protected for more than 400 years [2]. Dominant species of the BF are *Castanopsis chinensis* Hance, *Schima superba* Gardn. & Champ., *Cryptocarya chinensis* (Hance) Hemsl., *Machilus chinensis* (Champ. ex Benth.) Hemsl. and *Syzygium rehderianum* Merr. & Perry in the canopy and subcanopy layer [2], [4]. The other two forests in DHS (MF and PF) were clear-cut in the 1930s, and *P. massoniana* plantations were established thereafter. The pine plantation was naturally colonized by broadleaf species to become a mixed forest in MF. Meanwhile, the understory vegetation and litter in PM were harvested constantly until the late 1990s, which resulted in a dominance of *P. massoniana* (more than 90% of the total basal area) in PM. In MF, *Pinus massoniana* and *Schima superba* are the dominant tree species. The soils in the DHS are lateritic red earths (Oxisols) formed from sandstone [2], [4]. In HS site, the other two forests, i.e., a planted *Acacia auriculiformis* forest (AA), and a planted *Eucalyptus urophylla* forest, are located. Both AA and EU are 34 years old at the time of sampling. Previous studies reported that the soils in the two plantations are classified as Acrisols [5]. The annual precipitation and mean annual temperature are 1927 mm and 21.0 °C, respectively, in DHS [6], and 1580 mm and 22.5 °C, respectively, in HS [7].

### 2.2 Sampling

**Fig. 2:**
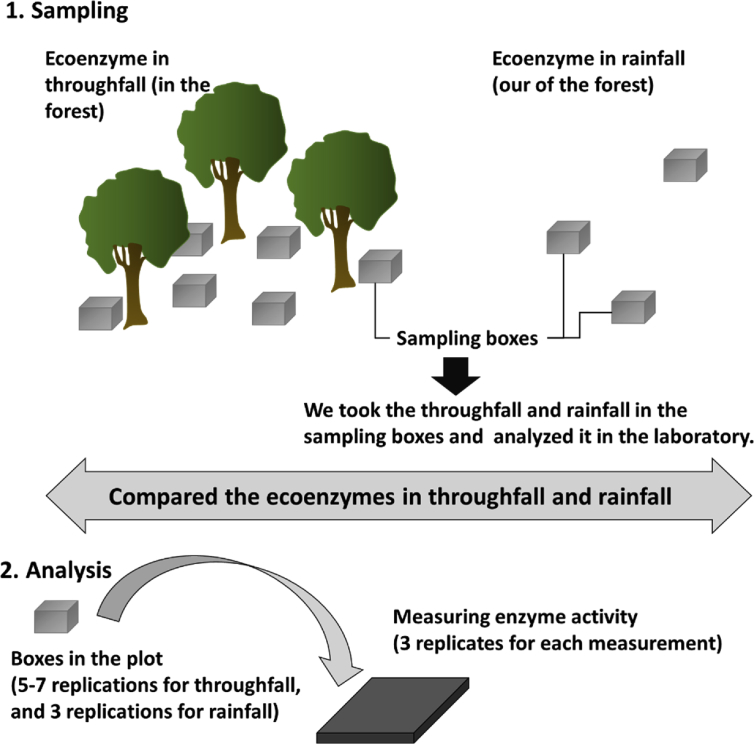
Research prosedures.

In August 2017, throughfall samples were collected in the five subtropical forests. We placed plastic boxes both inside and outside of the five forests (Fig. 2). We prepared 7, 5, 7, 6, and 6 replications of the boxes in BF, MF, PM, AA, and EU, respectively. We also collected rainfall samples in order to determine the background enzyme activity of rainfall. The boxes were placed at the outside of the forests, where there was no vegetation above the boxes. Rainfall samples were collected in triplicate in each research station. In total, 31 throughfall samples and 6 rainfall samples were collected. Those samples were collected as soon as possible after a rainfall event and kept cool/frozen in the research station near the forests.

### 2.3 Enzyme assay

We measured six types of ecoenzymes in the throughfall and rainfall samples. The activities of β-1,4-glucosidase (BG, EC 3.2.1.21), β-d-cellobiosidase (CBH, EC 3.2.1.91), β-1,4-N-acetyl-glucosaminidase (NAG, EC 3.2.1.52), leucine amino peptidase (LAP, EC 3.4.11.1), polyphenol oxidase (PPO, EC 1.10.3.2), and phosphomonoesterase (PME, EC 3.1.3.2) were determined. Fluorescence enzyme assays were used for determining hydrolytic enzyme activities [8], [9]. A portion of the collected samples (100 μ) was dispensed into 96-deep-well plates. For analyzing BG, CBH, NAG, and PME activities, substrates labeled with 4-methylumbelliferone (MUB) were added. For the analysis of LAP activity, 7-amino-4-methylcoumarin (MUC) were added. The final substrate concentration was 150 μM for BG, CBH, NAG, and PME, and 100 μM for LAP. The solution volume was 1000 μL. After mixed well, the deep plates were incubated for 4 hours at 20 °C in the dark. After the incubation, 250 μL of the incubated solution was transferred into black 96-well plates. We measured fluorescence (365 nm excitation, 450 nm emission) with a microplate spectrophotometer. Standard lines were prepared for all samples by determining the fluorescence of known concentrations of the MUB or MUC solutions with 100-μL aliquots of the collected samples. PPO activity was measured by spectrophotometric assay [9]. A portion of the collected samples (100 μ) was dispensed into 96-deep-well plates with 700 μL of pure water and 200 μL of dihydroxyphenylalanine (DOPA, 25 mM). The deep-well plates were incubated for 65 hours at 20 °C in the dark. Absorbance at 450 nm was measured using a microplate spectrophotometer. We prepared negative and blank controls for all enzyme assays. For minimizing “well to well variation” (Bell et al., 2013), three assay replicates in each plate were prepared. The activities of each types of ecoenzymes are represented in units of μg substrates (MUB for BG, CBH, NAG, and PME; MUC for LAP; and DOPA for PPO) h^−1^ L^−1^.
